# Review of Human Pathogenic Fungi: Molecular Biology and Pathogenic Mechanisms

**DOI:** 10.3389/fmicb.2015.00082

**Published:** 2015-02-11

**Authors:** Joshua D. Nosanchuk

**Affiliations:** Department of Medicine (Division of Infectious Diseases) and Department of Microbiology and Immunology, Albert Einstein College of MedicineBronx, NY, USA

**Keywords:** fungi, mold, yeast, molecular biology, pathogenesis, animal models

The complex interplay between host and microbe is especially evident in the pathogenesis of fungal diseases. Certain fungi, like *Candida albicans*, are commensals whereas others, like *Cryptococcus neoformans*, are environmental opportunists who have, in part, enhanced their capacity to cause devastating disease through interactions (e.g., a “boot camp”) over the millennia with environmental predators, such as amoebae (Casadevall et al., 2003). Moreover, the invading eukaryotic fungal cells have enormous similarities to mammalian cells, which significantly complicate therapeutic approaches to combat fungal diseases, which most frequently occur in hosts with compromised immunity. Human Pathogenic Fungi: Molecular Biology and Pathogenic Mechanisms, edited by Derek J. Sullivan and Gary P. Moran from Trinity College Dublin, provides a new collection of chapters that carefully delineate critically important aspects of the pathobiology of major human pathogenic fungi while concomitantly detailing recent significant advances in methods for their study and carefully reviewing relevant current epidemiological, clinical, and basic science information (Sullivan and Moran, 2014). The up-to-date, well-presented chapters serve to make this text a valuable reference for experts in the field as well as those new to fungal pathobiology. The authors contributing chapters to the book include well-known leaders in fungal pathogenesis, and several chapters have co-authors who are young, rising stars in their respective areas.

The editors divide the book into two interconnected sections. In Part I, the chapters focus on recent advances in approaches to studying fungal pathogenesis. In Part II, specific fungi are separately presented with a particular emphasis on the current knowledge of their cellular and molecular biology. Each chapter is carefully edited and easy to read. A real strength of the publication is that the chapters are highly linked to each other, but also stand as independent, extremely useful reviews of their individual, dedicated topics. An additional exceedingly useful aspect of this text is the carefully collected reference sets for each chapter, which will be invaluable especially to those new to the study of pathogenic fungi.

In Part I, there are six well-developed chapters that provide detailed information on the well-established and cutting edge methods for studying fungal pathogenesis. Chapters one and two discuss approaches for high-throughput sequencing and how analyses of these data translate into fundamental insights into fungal pathobiology, including the evolution of factors associated with virulence. This rich array of established and emerging data also facilitates system-wide, highly complex assessments of host-fungal interactions, and the application of systems biology to these data sets is carefully detailed in the third chapter. Building on concepts in the first three chapters, the fourth chapter describes exploration of pathogenesis through transcriptomic analyses. Validated animal models for studying fungal diseases are presented in the fifth chapter, including using these models to evaluate therapeutics. The final chapter of Part I, chapter six, uses *C. albicans* as a model organism to provide an overview of host responses to fungal cells, concomitantly looking at the roles of both the fungi and the host in these responses.

The pathobiology of specific fungi or groups of fungi is presented in separate chapters in Part II of Human Pathogenic Fungi: Molecular Biology and Pathogenic Mechanisms. The impact of secreted enzymes, the capacity to form biofilms, the ability to scavenge metals, and diverse other virulence attributes are discussed in these chapters. The seventh chapter dovetails well with the host response chapter preceding it, as it focuses on the recent advances in our knowledge of the clinical relevance and pathogenesis of *Candida.* Chapter eight discusses the various attributes that converge to provide *Aspergillus fumigatus* with the potent to cause devastating disease in immunocompromised hosts as well as allergic manifestations in immunologically intact individuals. *C. neoformans* is the focus of chapter nine, and I especially enjoyed reading the descriptions of capsular dynamics and plasticity as well as the relatively new finding that the fungus produces “titan” or “giant” cells during infection that are up to 100 μm in diameter (Zaragoza and Nielsen, 2013). Dermatophytes typically cause superficial disease, but are extremely common and, thus, economically important. Chapter ten describes the factors that contribute to the virulence of these species as well as presents new information gained through the sequencing of genomes from seven different dermatophyte species. The clinical importance and pathogenesis *Histoplasma capsulatum* and *Blastomyces dermatitidis* are rigorously explored in chapters eleven and twelve, respectively. The complexities of studying *Pneumocystis jerovecii* (i.e., it only grows in humans) and the significance of pneumocystosis are detailed in chapter thirteen. The final chapter, chapter fourteen, is co-authored by six Brazilian scientists who summarize the current knowledge regarding the virulence characteristics and pathogenesis of *Paracoccidioides.*

In sum, this well-presented work is a highly useful addition to the literature. The approach of presenting the current knowledge on the molecular and cellular biology of these important pathogens in conjunction with concise discussions of epidemiology, clinical manifestations, and therapeutic approaches, both current and potential, provides the reader with a thorough fundamental understanding of the particular aspect of each chapter. The extremely readable nature of the well-edited text will certainly facilitate its use by graduate students entering into the fields of fundamental or medical mycology and established researchers will appreciate the crisp reviews and their thorough references.

## Conflict of interest statement

The author declares that the research was conducted in the absence of any commercial or financial relationships that could be construed as a potential conflict of interest.
